# Wider Dental Care Coverage Associated with Lower Oral Health Inequalities: A Comparison Study between Japan and England

**DOI:** 10.3390/ijerph17155539

**Published:** 2020-07-31

**Authors:** Kanade Ito, Noriko Cable, Tatsuo Yamamoto, Kayo Suzuki, Katsunori Kondo, Ken Osaka, Georgios Tsakos, Richard G. Watt, Jun Aida

**Affiliations:** 1 Department of Oral Care for Systemic Health Support, Health Sciences and Biomedical Engineering, Graduate School of Medical and Dental Sciences, Tokyo Medical and Dental University, Bunkyo 113-8510, Japan; 2 Department of Epidemiology and Public Health, University College London, London WC1E 7HB, UK; n.cable@ucl.ac.uk (N.C.); g.tsakos@ucl.ac.uk (G.T.); r.watt@ucl.ac.uk (R.G.W.); 3 Department of Disaster Medicine and Dental Sociology, Graduate School of Dentistry, Kanagawa Dental University, Yokosuka 238-8580, Japan; yamamoto.tatsuo@kdu.ac.jp; 4 Department of Policy Studies, Aichi Gakuin University, Nisshin 470-0195, Japan; ksuzuki@psis.agu.ac.jp; 5 Department of Social Preventive Medical Sciences, Center for Preventive Medical Sciences, Chiba University, Chiba 260-0856, Japan; kkondo@chiba-u.jp; 6 Department of Gerontological Evaluation, Center for Gerontology and Social Science, National Center for Geriatrics and Gerontology, Obu 474-8511, Japan; 7 Department of International and Community Oral Health, Tohoku University, Sendai 980-8575, Japan; osaka@m.tohoku.ac.jp (K.O.); j-aida@umin.ac.jp (J.A.)

**Keywords:** international comparison, edentulism, oral health inequality, universal health coverage, slope index of inequality, relative index of inequality

## Abstract

Countries with different oral health care systems may have different levels of oral health related inequalities. We compared the socioeconomic inequalities in oral health among older adults in Japan and England. We used the data for adults aged 65 years or over from Japan (*N* = 79,707) and England (*N* = 5115) and estimated absolute inequality (the Slope Index of Inequality, SII) and relative inequality (the Relative Index of Inequality, RII) for edentulism (the condition of having no natural teeth) by educational attainment and income. All analyses were adjusted for sex and age. Overall, 14% of the Japanese subjects and 21% of the English were edentulous. In both Japan and England, lower income and educational attainment were significantly associated with a higher risk of being edentulous. Education-based SII in Japan and England were 9.9% and 26.7%, respectively, and RII were 2.5 and 4.8, respectively. Income-based SII in Japan and England were 9.2% and 14.4%, respectively, and RII were 2.1 and 1.9, respectively. Social inequalities in edentulous individuals exist in both these high-income countries, but Japan, with wider coverage for dental care, had lower levels of inequality than England.

## 1. Introduction

Health care systems are one of the structural social determinants of health inequalities [1]. There are substantial differences in the health care system between countries in terms of the coverage for health care across populations, for example, the target person, the scope of treatment available, the target disease and the copayment [2,3,4]. Universal health coverage has been promoted in the Millennium Summit at the United Nations and World Health Organisation (WHO), and expanding oral care in universal health coverage is desired [5,6].

There are differences in oral health care coverage across countries. Japan and England have a similar universal health care system. However, the dental health care system was the difference between the two countries [7,8]. Japan has a public universal health care insurance system that includes most of dental treatments. As a result, Japan is one of the countries providing the most accessible dental care in the world from an international perspective. Japanese people attend dental care 3.2 times per year (reported in 2011), which is the most frequent dental attendance pattern among the Organisation for Economic Co-operation and Development (OECD) countries [9]. The percentage of Japanese household expenditure on dental services is 0.4% [10]. In England, some dental care is also covered by the public health care system, the National Health Service (NHS) [8]. In the United Kingdom (UK), dental care in the past year was 0.8 times per person (reported in 2011) [9], and the percentage of household expenditure on dental services was 12.0% [11]. These large differences in health care characteristics and coverage could affect the level of inequalities in oral health at the population level. In both Japan and England, there are inequalities in access to dental care [12,13,14,15,16,17]. Recently, universal health coverage is promoted in the world, and it is considered to decrease oral health inequalities [1]. However, few studies compared the degree of oral health inequalities between the countries with different coverages, and to the best of our knowledge, no international comparative study on oral health inequalities has included Japan, the country with one of the highest oral health care coverage.

The global burden of disease study reported the prevalent nature and large burden of severe tooth loss [18]. Edentulism (the condition of having no natural teeth) is the final form of severe tooth loss. Accumulation of the experiences of dental caries and periodontal disease through life-course cause edentulism [19,20]. Therefore, it is prevalent among the older population [21], and it can reflect oral health inequalities determined by a wider range of dental health care across the life-course. We hypothesised that inequalities in edentulism would be smaller in Japan with wider dental care coverage than in England. The objective of this study is to compare the association between socioeconomic status and edentulism among older adults in Japan and England.

## 2. Materials and Methods 

### 2.1. Participants

**Figure 1:**
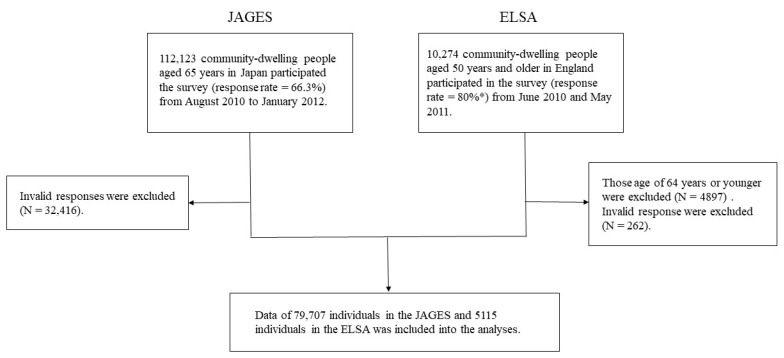
Flowchart of the selection of participants for the study (JAGES, Japan Gerontological Evaluation Study; ELSA, English Longitudinal Study of Ageing). * This response rate was taken from cohort profile of ELSA [23].

We used cross-sectional data from the Japan Gerontological Evaluation Study (JAGES) [22] and the English Longitudinal Study of Ageing (ELSA) [23]. The JAGES and ELSA are both ongoing prospective cohort studies investigating social and behavioural factors associated with a functional decline or cognitive impairment among older individuals. The target population of the JAGES was 169,215 community-dwelling people aged 65 years and older selected from 31 municipalities in 12 prefectures in Japan from August 2010 to January 2012 (Wave 3). A total of 112,123 people participated in the JAGES (response rate = 66.3%). The ELSA data were collected between June 2010 and May 2011 (Wave 5) from a total of 10,274 community-dwelling people aged 50 years and older living in England. Those certified for long-term care in JAGES and those under the age of 65 years in ELSA were excluded. Then, both JAGES and ELSA data obtained from older adults aged over 65 years and older with valid responses (no missing data) in the variables included in this study were used for our analyses (Figure 1). 

### 2.2. Ethical Considerations

Ethical approval for the study was obtained from the Ethics Committee at Nihon Fukushi University, Japan (Approval number: 10–05). ELSA was approved by the South Central Berkshire Research Ethics Committee through an application to the National Research Ethics Service [24]. 

### 2.3. Dependent Variable

The dependent variable for the present analysis was edentulism (= “Has no teeth”). The JAGES self-report questionnaire on the current dental status of the participants used the following question: “How many natural teeth do you presently have?” There were four choices: (1) I have 20 or more natural teeth, (2) I have 10 to 19 natural teeth, (3) I have 1 to 9 natural teeth, or (4) I have no natural teeth. We categorised the answers as “dentate” (1–3) and answer “edentulous” (4). In the ELSA, the question was as follows: “In relation to your dental health, which of the following applies to you?” There were four choices: (1) Has no natural teeth and wears dentures, (2) Has both natural teeth and denture(s), (3) Has only natural teeth, or (4) Has neither natural teeth nor dentures. We categorised the answers as “dentate” (2 and 3) and “edentulous” (1 and 4).

### 2.4. Explanatory Variables

We used equivalised annual income and educational attainment as indicators of participants’ socioeconomic status. Income levels were classified as follows: lowest, low, middle and high (JAGES: ≤12,500, 12,501–19,445, 19,446–30,619, ≥30,620 (USD, 1 USD = 100 JPY), ELSA: ≤11,911, 11,912–<16,459, 16,459–<23,810, >=23,810 (USD, 1 USD = 0.8 GBP), respectively). For educational attainment, we acknowledge that possible country differences in the education system between Japan and England. We consulted with the ELSA data management team and harmonised the education level between the two countries (ELSA 3 and 4 Label = JAGES 1 and 2 (15 years or younger), ELSA 5, 6 and 7 Label = JAGES 3 (16–18 years) and ELSA 8 Label = JAGES 4 (19 or older)). The samples were categorised according to the age at which they completed their formal education as low (15 years or younger), middle (16–18 years) and high (19 years or older) for both the JAGES and ELSA. Age (65–69, 70–74, 75–79, 80–84 and ≥85 years) and sex (men and women) were used as covariates. 

### 2.5. Data Analyses

Firstly, we described the demographic characteristics of the participants. For the participants’ age, we used the median and first quartile–third quartile because our data were not distributed normally (Kolmogorov–Smirnov test, *p* < 0.001). Secondly, to show the sex- and age-adjusted association of socioeconomic status on edentulism, sex- and age-adjusted prevalence ratios were calculated from Poisson regression analyses. Finally, we estimated the Slope Index of Inequality (SII) and the Relative Index of Inequality (RII) to evaluate absolute and relative inequalities [25] in edentulism. SII referents the absolute difference in a health indicator between those with a higher and those with a lower level of socioeconomic status. RII is a relative measure of inequality that indicates the ratio of health status between the higher and lower levels of socioeconomic status groups. Both indicators were calculated using regression-based methods [26]. To determine the explanatory factors in the association between socioeconomic status (income and educational attainment) and edentulism, we built the models for each country as follows. Model 1 tested the association between a socioeconomic status variable (income or educational attainment) and edentulism (univariate model). Model 2 examined the association between a socioeconomic status variable (incomes or educational attainment) and edentulism after adjusting for age and sex (age and sex adjusted model). All analyses were done by Stata MP version 14 [26]. 

## 3. Results

We used the data obtained from 79,707 individuals in the JAGES and 5115 individuals in the ELSA Wave that had no missing responses. There were 49.6% men in the JAGES and 45.6% men in the ELSA. The median (first quartile–third quartile) age was 73.0 (69.0–77.0) years for JAGES and 73.0 (69.0–79.0) years for ELSA. The prevalence of edentulism was 13.8% for JAGES and 20.6% for ELSA.

Table 1 shows the prevalence of sociodemographic attributes according to the dental status from JAGES and ELSA. Women had a higher proportion of edentulism than men, especially in ELSA. Social gradients in dental status were observed in both samples, and participants with higher education or income had lower percentages of edentulism.

**Figure 2:**
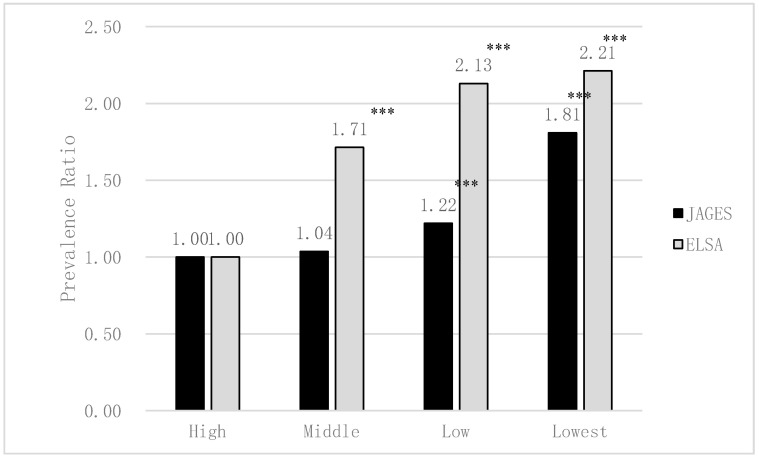
The association between edentulism and equivalised annual income in Japan and England estimated by Poisson regression analyses (adjusted for sex and age; JAGES *N* = 79,707, ELSA *N* = 5115). *** *p* < 0.001.

**Figure 3:**
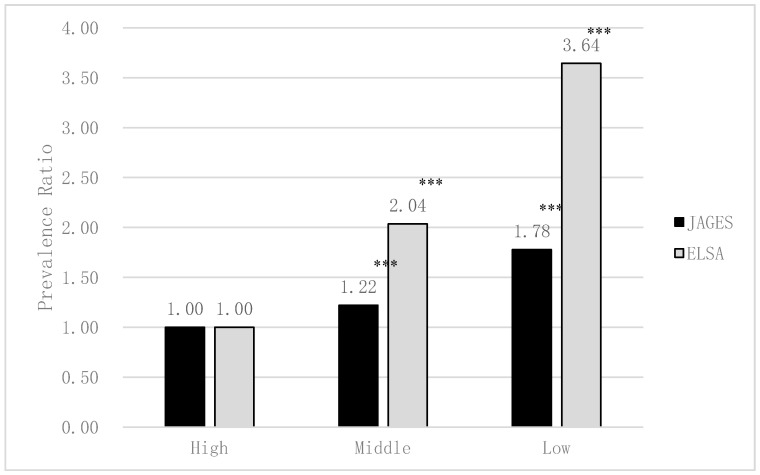
The association between edentulism and educational attainment in Japan and England estimated by Poisson regression analyses (adjusted for sex and age; JAGES *N* = 79,707, ELSA *N* = 5115). *** *p* < 0.001.

Figure 2 and Figure 3 show the association between edentulism and socioeconomic status in Japan and England. In both Japan and England, lower income and lower educational attainment were significantly and independently associated with both higher risk of edentulism (excluding the middle income of Japan, *p* < 0.001). Income and educational attainment gradient were appeared to be greater in England than in Japan in edentulism.

Table 2 shows the SII and RII of edentulism in Japan and England. The absolute socioeconomic difference in edentulism between groups with the lowest and highest income (SII for income) was 11.8% (95% CI, 11.0; 12.0) for JAGES and 18.5% (95% CI, 15.0; 21.9) for ELSA (univariate models). After adjusting for age and sex, significant absolute income differences in edentulism for both JAGES and ELSA (JAGES = 9.2% (95% CI, 8.6; 9.9) and ELSA = 14.4% (95% CI, 11.0; 17.7)) remained. The relative income differences in edentulism (RII for income) were also significant after adjustment for age and sex for both JAGES and ELSA (JAGES = 2.1 (95% CI, 2.0; 2.2) and ELSA = 1.9 (95% CI, 1.6; 2.3)). 

For education-based inequalities, the absolute difference in edentulism between groups with the lowest and highest educational attainment (SII for education) was 15.0% (95% CI, 14.2; 15.8) for JAGES and 31.9% (95% CI, 28.2; 35.6) for ELSA. Even after adjusting for age and sex, significant absolute educational attainment differences in edentulism for both JAGES and ELSA (JAGES = 9.9% (95% CI, 9.2; 10.7) and ELSA = 26.7% (95% CI, 23.1; 30.2)) were observed. The relative educational attainment differences in edentulism (RII for education) were also significant after adjustment for age and sex for both studies (JAGES = 2.5 (95% CI, 2.3; 2.6) and ELSA = 4.8 (95% CI, 3.7; 6.2)).

Overall, absolute oral health inequalities were smaller in Japan than in England. Education appears to play a crucial role in determining oral health inequalities compared to income, particularly in England.

## 4. Discussion

The present study reports both absolute and relative socioeconomic (income and education) inequalities in edentulism in Japan and England. In general, oral health inequalities were smaller in Japan, a country with one of the highest levels of public coverage for dental care, compared to England. The prevalence of edentulism was higher in England than in Japan. Socioeconomic inequalities, especially educational inequalities, were relatively larger in England than in Japan, and the differences between the two countries were most obvious.

For oral health inequalities, our results were consistent with previous studies. Japanese national health data showed that lower educational attainment and lower equivalent household expenditure were associated with fewer remaining teeth [27], while lower income was associated with poor dental status in Japanese older people [28]. Similarly, socioeconomic inequalities, number of remaining teeth [29], edentulism [30], dental caries [31,32] and periodontal disease [33,34] were reported in the UK. Using large cohort data with harmonised measures, our findings of oral health inequalities determined by income and education offer extended support to previous studies.

Differences in accessibility to dental care could explain the present results of smaller oral health inequalities in Japan than in England. Inequalities in access to dental care are also reported in both countries [16,35]. However, in the UK, out-of-pocket expenses for dental care were higher, and the frequency of dental visits was lower than in Japan [9,10,11]. In England, previous studies showed that almost 20% of the participants tried to get an NHS dental appointment, but failed to get one within a reasonable time [36,37]. People unable to book an NHS dental appointment had to visit a costly private dentist instead. On the other hand, the Japanese insurance system provides a free access system to dental care, allowing the Japanese to select any medical institution where they can receive high-quality medical and dental services at a low cost [38]. Moreover, Japan has a life-course oral health care system [7]. This system is provided as part of general health services, and the programme is based on health legislation. For preschool children, a national programme that includes physical, medical and dental examinations is conducted for all 18-month-olds and 3-year-olds. After the dental examination, dental hygienists provide maternal and child oral health education. For school children, every public primary, junior and senior high school has a school dentist. The school dentist performs dental examination for the school children at least once a year. Moreover, all schools incorporate oral health education into their curriculum. These systems are believed to be associated with low oral health inequality in Japan.

The United Nations and WHO promote universal health coverage [5,39]. Our findings suggest the possibility that more comprehensive dental care coverage reduces oral health inequalities. A wider inclusion of dental care in universal health coverage is required to improve oral health and reduce oral health inequalities. Notably, even in Japan, there are still significant levels of oral health inequalities, suggesting room for improvement, such as a wide range of oral health promotion in the context of social determinants of health [6,40]. 

Our study had several limitations and strengths. First, our study used a cross-sectional design, and hence, we could not establish a causal relationship between socioeconomic indicators and edentulism. Second, the measurements were obtained from a self-administered questionnaire. However, clinical assessment of remaining teeth is more accurate than self-administered questionnaires. Still, the validity of the self-reported number of remaining teeth was verified [31]. Finally, potential biases could be likely due to fewer cases. However, the strength of this study was that it used large cohort data from each country with harmonised measures, offering the level of oral health inequalities in both absolute and relative terms that were comparable. 

## 5. Conclusions

Both absolute and relative inequalities in edentulism were found in Japan and England. Most of the inequality measurements suggested that oral health inequalities were smaller in Japan, a country with one of the highest levels of public coverage for dental care in the world.

## Figures and Tables

**Table 1:** Descriptive distribution of edentulous participants in Japan and England.

		Total	JAGES (*N* = 79,707)		Total	ELSA (*N* = 5115)	
			*N*	%		*N*	%
Age							
	65–69 year old	24,567	1331	5.4%	1537	172	11.2%
	70–74 year old	23,911	2322	9.7%	1411	244	17.3%
	75–79 year old	17,152	2904	16.9%	1029	239	23.2%
	80–84 year old	9506	2586	27.2%	640	190	29.7%
	85+ year old	4571	1881	41.2%	498	209	42.0%
Sex							
	Men	39,568	5491	13.9%	2334	417	17.9%
	Women	40,139	5533	13.8%	2781	637	22.9%
Income							
	Lowest	21,076	4513	21.4%	1278	356	27.9%
	Low	19,455	2386	12.3%	1280	322	25.2%
	Middle	18,916	2058	10.9%	1279	247	19.3%
	High	20,260	2067	10.2%	1278	129	10.1%
Educational attainment							
	Low	36,085	1934	21.0%	2815	779	27.7%
	Middle	28,603	714	10.5%	1645	233	14.2%
	High	15,019	297	8.2%	655	42	6.4%

JAGES, Japan Gerontological Evaluation Study; ELSA, English Longitudinal Study of Ageing.

**Table 2:** Absolute and relative socioeconomic inequalities of edentulousness in JAGES and ELSA.

		JAGES (*N* = 79,707)		ELSA (*N* = 5115)	
		Univariate Model	Age and Sex Adjusted Model	Univariate Model	Age and Sex Adjusted Model
Income	SII (%) (95% CI)	11.77 (11.04; 12.50)	9.24 (8.58; 9.90)	18.46 (15.02; 21.90)	14.35 (10.97; 17.73)
	RII (95% CI)	2.47 (2.34; 2.61)	2.07 (1.96; 2.18)	2.40 (2.03; 2.83)	1.92 (1.63; 2.27)
Educational attainment	SII (%) (95% CI)	15.00 (14.18; 15.83)	9.93 (9.19; 10.68)	31.93 (28.22; 35.64)	26.65 (23.11; 30.20)
	RII (95% CI)	3.36 (3.13; 3.61)	2.45 (2.29; 2.63)	5.88 (4.55; 7.61)	4.79 (3.70; 6.19)

SII, Slope Index of Inequality; RII, Relative Index of Inequality.

## Abbreviations

CI	Confidence Interval
ELSA	English Longitudinal Study of Ageing
OECD	Organisation for Economic Co-operation and Development
JAGES	Japan Gerontological Evaluation Study
NHS	National Health Service
RII	Relative Index of Inequality
UK	United Kingdom
SII	Slope Index of Inequality
WHO	World Health Organisation
